# Fostering the Resilience of People With Dementia: A Narrative Literature Review

**DOI:** 10.3389/fmed.2020.00045

**Published:** 2020-02-25

**Authors:** Sally Whelan, Áine Teahan, Dympna Casey

**Affiliations:** ^1^School of Nursing and Midwifery, National University of Ireland, Galway, Ireland; ^2^Centre for Economic and Social Research on Dementia, National University of Ireland, Galway, Ireland

**Keywords:** dementia, Alzheimer's, resilience, systematic review, aging, interventions

## Abstract

**Background:** Resilience is a process through which people use resources to adapt to adversity. Interventions aiming to support resilience in people with dementia have been developed. However, the optimal content, structure and impact of these interventions is unclear. This literature review explores the factors through which interventions foster resilience in people with dementia and examines their efficacy.

**Methods:** Eight databases were searched systematically, for literature published from 2000 to 2019. Following the removal of duplicate articles, the titles and abstracts of 6,749 articles were screened. Articles were selected if they: reported empirical studies in English; focused on resilience; involved people with dementia and psychosocial interventions. The full text of 53 articles were examined and three studies, reported in six papers, were included in the final review. Data were systematically extracted, and two authors critiqued the studies using the Critical Appraisal Skills Programme check lists. The studies were examined to determine how resilience was defined and operationalized and their findings were synthesized using the theoretical resilience framework.

**Results:** Five interventions aiming to foster resilience were identified: Dementia Advisors; Peer Support Network Services; Visual Arts Enrichment Activities; Memory Makers; and Early-Stage and Beyond Community Activities. All studies defined resilience as a process and most involved people with mild dementia who had family carers. The interventions impacted resilience by reducing the adversity of stigma and social isolation; increasing personal and social resources, providing stigma-free space and reciprocal support. Interventions empowered people with dementia, increasing their self-esteem and self-worth. Resilience can be fostered both during, and after interventions. However, the efficacy of interventions could not be determined because the research designs utilized did not measure efficacy.

**Conclusions:** Interventions need facilitators to ensure they are strength-based, person-centered and they enable reciprocal social interactions. Future research needs to develop interventions that aim to foster the resilience of people with dementia who lack family carers and/or have more advanced dementia through meaningful activities that are identified by people with dementia as important to their resilience. Robust methodologies, including randomized controlled trials should be used to measure effectiveness and explore the impact of interventions regarding the: interplay between individual and community resources; the importance of reciprocity; and temporal aspects of resilience.

## Introduction

Dementia is a chronic progressive syndrome, which currently affects 50 million people worldwide (1). Having dementia can negatively impact the person's cognitive functioning, memory, thinking, orientation, language, and emotional control (1). Dementia can cause anxiety (2), and it may be linked to depression (3). Every year, as more people live into old age, there are 10 million people newly diagnosed with dementia (1). Consequently, it is increasingly important to develop strategies that facilitate and support people with dementia to remain independent and functioning well for as long as possible (4).

Resilience is important for people with dementia (5) because it can help with the challenges of living with the condition (6, 7). Resilience has been defined as a dynamic “process of effectively negotiating, adapting to, or managing significant sources of stress or trauma” [(8), p. 2]. Resilience has also been described in terms of a resilience framework (9) which draws upon the ecological systems theory (10). This framework regards resilience as occurring within a complex interacting multi-layered system, in the presence of a significant adversity, which can be acute or chronic in nature (8, 11). A person's response to adversity is facilitated by use of, and access to, resources that can be internal and/or external to the individual in their environment. There are a range of possible resilience outcomes, from vulnerability to flourishing (12). Outcomes of resilience can include maintaining normal development or competence in the presence of mental or physical health difficulties (9). Therefore, resilience can be present when a person with a chronic disease adapts to the condition and demonstrates processes that include acknowledging the condition, gaining a sense of control over it and integrating it into their life and lifestyle (13).

In the context of dementia, resilience is complex and multifaceted (4). It involves the use of resources to negotiate living with the challenges of dementia (14) and the compensatory practices of other people who are close to the individual with dementia (significant others), who act as a resource to support the person, as the dementia progresses (4). Resilience in dementia is strongly related to being socially connected with other people (15) and the participation of individual people with dementia in purposeful activity (16). Harris (17) applied the theoretical framework of resilience using in-depth case study methodology and the qualitative interviewing of people with dementia (*n* = 2) who were “doing okay” and managing to live well with their dementia. Harris (17) found that positive adaptation in dementia involved overcoming negative influences and having assets and protective factors that outweighed the risks and vulnerabilities experienced by individuals with dementia. They identified that in dementia assets included: having effective coping strategies; acceptance of the dementia diagnosis; accepting changes to life and the need to accept help from available support networks; a positive attitude; and productivity. Whereas, protective factors included: positive relationships with other people that supported personhood (18); and having positive role models. Other researchers have also emphasized the importance of acceptance (19) and of having positive thoughts and feelings (20). In addition, resilience in dementia has been characterized as a process of continual adjustment through which people with dementia learn to live with progressive limitations in their lives (21, 22).

Core outcome sets (23) for resilience in dementia have not yet been established but there has been a small amount of research focusing on outcome measures. Stoner et al. (24, 25) developed and validated with people with dementia (*n* = 126), the Positive Psychology Outcome Measure (PPOM) which measures capacity for resilience, and hope. PPOM has to our knowledge yet to be utilized in research, but Stoner et al. (25) found that PPOM may assist with the future development of asset–based approaches and interventions for dementia. From this literature, and that described above, it can be determined that the capacity of people with dementia for resilience can be improved through the presence of protective factors and that outcomes for resilience in dementia include: having capacity for resilience and protective factors; having the ability to cope effectively and recover from stress; having the ability to adjust and adapt attitudes and behavior to respond positively to dementia; and the ability to accept the challenges and limitations of life with dementia.

Psychosocial interventions aiming to support resilience in people with dementia need to be informed by factors that support and limit resilience (4). However, to date no published literature has examined the existing evidence concerning the content, structure and impact of interventions that aim to support resilience in people with dementia. This narrative literature review aims to explore the evidence concerning interventions that aim to foster resilience in people with dementia: to identify and examine how the concept of resilience is defined and operationalized in these investigations, the efficacy of interventions and the factors through which they impact resilience.

The objectives of this research were to:

- Identify and describe the psychosocial interventions designed to foster the resilience of people with dementia.
- Describe how the interventions were perceived and experienced by people with dementia.
- Critically appraise the methodologies used to design and investigate the interventions.
- Apply the empirical findings of the studies reviewed to the resilience process and framework.
- Describe the efficacy and impact of the interventions on the resilience process of people with dementia.
- Examine the factors that impacted the effectiveness of the interventions.

## Methods

### Search Strategy

A comprehensive and systematic search of the literature published from 2000 to 2019 was conducted with the guidance of an expert librarian. Eight databases: Scopus, Web of science, EBSCO-CINAHL, Ageline, PsycINFO, Cochrane, OpenGrey, and Proquest were utilized. Abstracts and titles were searched using keywords, MeSH terms and subject headings (Table 1), which were selected as they corresponded to the key characteristics of resilience in dementia that have been described above. An example of the search strategy outcomes is provided in Appendix i (Supplementary Material). In addition, the references of relevant papers were hand searched and their citations were examined using Google Scholar.

**Table 1:** Search terms.

**Population**	**Phenomenon of interest**	
Dementi^*^ OR Alzheimer's Disease OR Alzheimer^*^ OR Lewy body OR (Korsakoff OR Creutzfeldt-Jakob) N2 (disease OR syndrome) OR “Creuzfeldt-Jakob Disease” OR Frontotemporal dementia OR Huntington's Disease (Mixed OR Vascular) N2 dementia OR Parkinson's Disease	Resilien^*^ OR Adapt^*^ OR “Bounce back” OR accept^*^ OR Cop^*^ OR Adjust^*^ OR “protective factors”	Intervention^*^ OR Improve^*^ OR enhance^*^ OR increase^*^ OR therap^*^ OR promot^*^ OR foster^*^ OR program^*^ OR support^*^ OR treat^*^ OR educ^*^ OR mang^*^ OR method^*^ OR approach^*^ OR strategy^*^

* *All possible endings of this word were included in the search*.

### Inclusion and Exclusion Criteria

Papers were screened for eligibility by SW, the lead author. To be included, items needed to report empirical studies that involved people with dementia with any type of dementia of any severity. Studies also needed to involve non-pharmacological psychosocial interventions that addressed resilience or where this was named as an outcome measure. Interventions were defined as any physical, cognitive or social activities that aimed to maintain or improve “functioning, interpersonal relationships and well-being in people with dementia” (26). All comparators to the interventions were included: treatment as usual, no-treatment control, comparison with other interventions, usual treatment/care as were all design methods. Studies were excluded if they involved non-psychological interpretations of resilience, such as resilience in relation to the physical health or the geographical environment, and if they involved people with mild cognitive impairment or involved pharmacological interventions. They were also excluded if the studies used proxy terms for resilience such as self-efficacy, sense of coherence, hardiness, or quality of life. This ensured that the review focused on interventions which explicitly aimed to foster resilience.

### Data Extraction and Quality Assessment

Data from the selected papers were extracted systematically, by SW, using an extraction form relevant to the research objectives. This form captured the key features of the included studies (Table 2). As critical appraisal of studies has been strongly recommended when performing narrative reviews (33–35), the methodological strengths and limitations of the studies were assessed independently by two reviewers (SW, ÁT) using the Critical Appraisal Skills Programme (CASP-uk.net) qualitative checklist. The CASP checklist is a widely used tool for qualitative evidence synthesis and is recommended by World Health Organization guidelines (36). No study was excluded as a result of this quality assessment.

**Table 2:** Key features of the studies.

**Study ID Country Overall design**	**Aim**	**Intervention name Target Population Facilitators**	**Intervention description**	**Context delivered, duration, frequency**	**Study design and methods**	**Study population**	**Results**	**Conclusion**
Clarke et al. (27, 28) UK	To compare the influence of DA and PSN services to identify ways they contribute to well-being and resilience of people with dementia and family carers	1. DA 2. PSN People with dementia and Carers/FamiliesLay Health Workers	1. Signpost to other services and ongoing support. Lay Health Worker 2. Psychosocial Support in Alzheimer Society support groups and dementia cafes.	Community Ongoing	Mixed Methods Qualitative semi-structured Interviews Thematic analysis Well-being and QoL surveys using ASCOT and DEMQoL. Statistically analyzed.	People with dementia (*n* = 47) Early Dementia, family carers (*n* = 54), staff and stakeholders (*n* = 82).	Themes -Addressing the needs of the individual and community -Promoting independence. -Control and choice. -Getting a life back.	Public health models of healthcare provision. Should be used to promote resilience.
Newman et al. (29) and Windle et al. (30) UK	To evaluate the impact of visual arts enrichment activities on opportunities for resilience.	Visual arts enrichment activities people with dementia. Artists trained about dementia	Creative individual and collective activities	Care Home Weekly, 2 h for 3 months	Mixed Methods (only Qualitative data focused on resilience) Interviews baseline, post-intervention, and 3 months follow up with People with dementia, relatives, and Carers. Sessions Videoed Facilitator Structured notes.	People with dementia (*n* = 48) in care homes (*n* = 4) aged 70 to 99, CDR scale—*n* = 6 was 0.5 questionable; *n* = 18 1 mild; *n* = 8 2 moderate; *n* = 16 3 severe, care staff, family (*n* = 37)	Supported resilience through creative expression, increased communication, improved self-esteem and relationships with significant others.	Resilience can be supported by visual arts enrichment activities. The concept of respondent habitus may be useful.
Matchar et al. (31) USA	(Not explicitly stated)	Early-Stage and Beyond Community Post Memory Makers People with dementia and Family Carers Four Masters level Social Workers trained by Alzheimer's Association Early-Stage Group Facilitators Manual.	1. Lunch gatherings 2. Museum tours, activities, lunch 3. Support groups 4. Workshop for partners 5. Carer support groups 6. Lecture series for carers 7. Concerts, movies, education	Community 1. Monthly 2. Monthly 3. Monthly 4. 4 monthly every 1–2 years 5. Monthly 6. Quarterly 7. Random	Observational and Descriptively reported rather than using specific outcome measures.	Graduates from 16 Memory Makers support programme groups Family units (*n* = 1,799) with people early dementia (*n* = 166; aged 49–93) and their care givers (*n* = 178).	Resilience fostered through acceptance, disclosure, significant others, sense of purpose, routines, and familiar environments and memory aids, showing up/value of a support group, faith.	Resilience is of critical importance to people with dementia regarding acceptance of diagnosis and adaptation to it and there is limited work completed to date as to how resilience can be strengthened.
Matchar and Gwyther (32) USA	To explore the impact on resilience of an Alzheimer education and support group	Memory Makers program Structured Educational support group People with dementia and Family Carers 2 Masters level Social Workers trained by Alzheimer's Association Early-Stage Group Facilitators Manual.	Structured Educational support group; with carer-people with dementia 5–12 dyads. 75 min of discussion separately and then dyads together on different topics weekly.	Community 3 h weekly, for 8 weeks	Observational Descriptive Evidence from 4 groups. Open-ended evaluation surveys were emailed after intervention. Anecdotes from these combined with facilitator observations	People with early dementia and care partner dyads (*n* = 35) spouse 86% adult daughter 14%	People with dementia expressed gratitude for care partner, perceived small victories sustained their resilience. Groups shared coping strategies, expressed hope, humor, living the best lives they could, reciprocal caring.	Resilience benefits from sense of belonging to peer group.

## Review Findings

**Figure 1:**
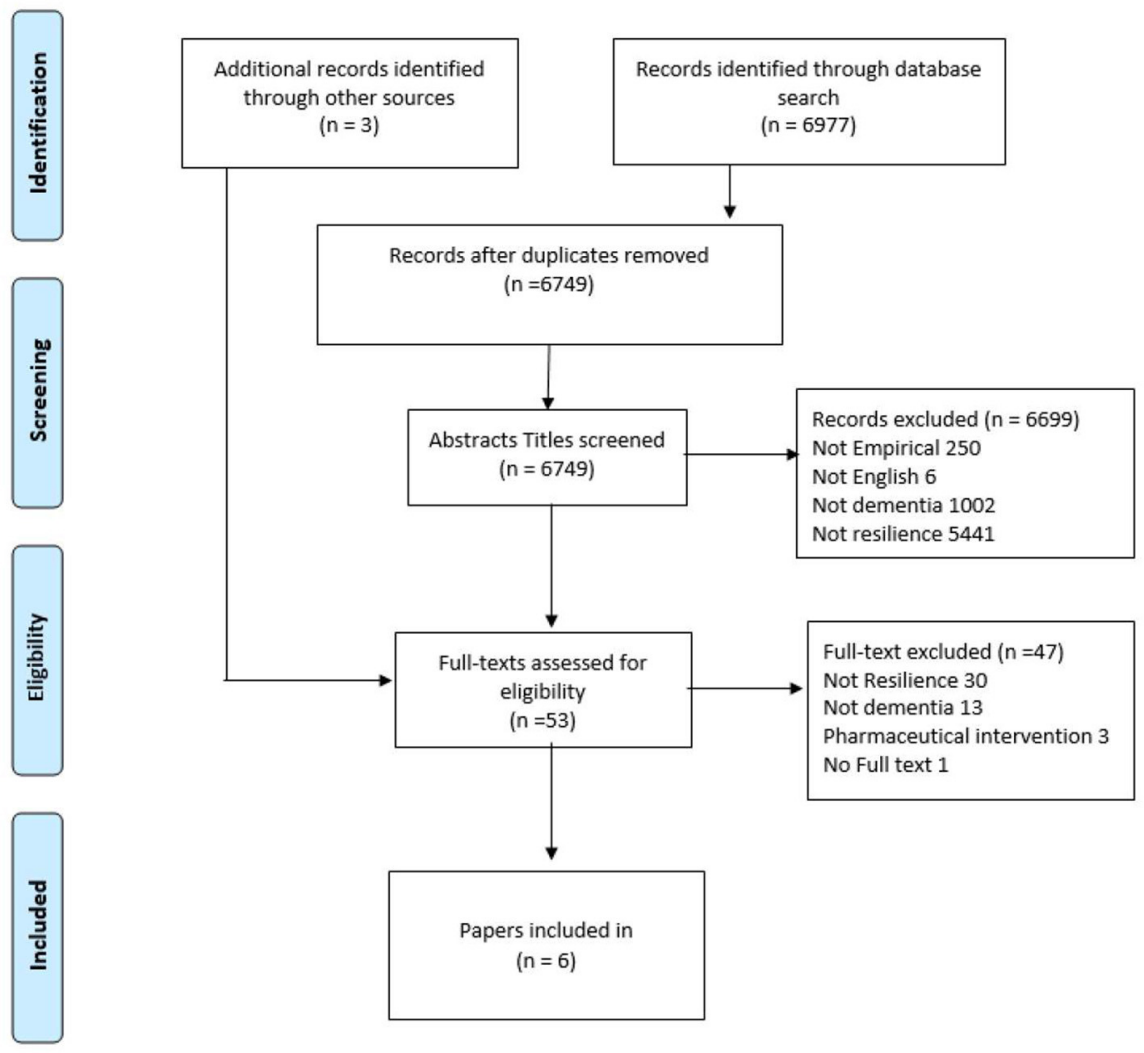
Prisma flow diagram describing the identification, screening, eligibility, and inclusion criteria of the studies identified under the scope of this review.

The PRISMA diagram in Figure 1 summarizes the selection and screening process (37). The initial search identified 6,977 items. After removing duplicates, the abstract and titles of 6,749 items were screened according to the inclusion and exclusion criteria. Three additional papers were identified through hand searching the reference lists of relevant studies. This resulted in 53 studies being retained for full-text review, against the inclusion criteria. The final review included six papers that reported five interventions (27–32).

An overview of the studies and the interventions is provided below. Enough detail is provided in this overview to enable readers to make sense of studies' context and findings (33), as has been strongly recommended in narrative literature reviews (34, 35). Following the overview, this review then focuses on how the concept of resilience was defined and operationalized within the included studies. After this, the findings of the studies are interpreted in relation to the resilience framework (9).

### Overview of the Studies and the Interventions for Resilience

#### Dementia Advisors and Peer Support Network Services

Clarke et al. (27, 28) conducted a study which evaluated a national programme in the UK that aimed to compare the influence of dementia advisers (DA's) and peer support network (PSN) services on the well-being and resilience of people with dementia and their family carers, living in a community setting. The DA's provided information and an ongoing point of contact for service users. They aimed to provide information about dementia and signpost other services, such as social groups, legal or financial supports. The PSN provided emotional and social support to people with dementia and carers through Alzheimer Society support groups and dementia cafes. Both DA's and PSN facilitators were lay health workers, and many were volunteers (28). At the time of the Clarke et al.'s evaluation, the interventions had been operating for 10 years at 40 demonstrations sites.

Clarke et al. used a mixed methods design which emphasized qualitative methodology (28). An organizational survey was conducted along with case studies of some demonstration sites (*n* = 8). People with dementia were interviewed, at their convenience, alone or with their family carers 1–3 times. These semi-structured interviews lasted up to 2 h. At the time of interview, quantitative questionnaires were also administered. These included the adult social care outcomes toolkit (ASCOT) (38) which collected data on unmet needs and the DEMQoL (39), that recorded health related quality of life. In addition, staff and stakeholders (*n* = 82) were interviewed. Participants were recruited through key staff working at the chosen demonstration sites and a sampling matrix was used to select a range of staff and stakeholders who had accessed the services. Participants included family carers (*n* = 54) and people with dementia (*n* = 47), the majority of whom were aged 65–85 years and had early stage (mild) dementia. The quantitative data were analyzed using SPSS, to ascertain statistical representation of frequency and modal responses for each respondent and all people with dementia as a group. All the interview data was uploaded into NVIVO and descriptive content analysis was conducted on 25 of the interview transcripts from which the research team developed a coding framework which was used to analyse the remaining data into themes.

Clarke et al. identified three themes: addressing the needs of individual and communities; promoting choice, control and independence; and getting a life back. The findings included in the first theme revealed that both interventions operated through identifying and responding to the needs of their users. The DA and PSN were informed and shaped by the needs and expressed desires of the people with dementia and their carers. The carers wished to remain well and both the carer and person with dementia wished that the stigma surrounding dementia could be reduced. The data also revealed that the PSN and DA's responded to the needs of the people with dementia by providing a wider range of services than those offered by traditional providers, including for example, gardening clubs and music groups. Also, the PSN and DA facilitators raised awareness about dementia with the wider public through providing training and information. This was illustrated by a carer who said:

‘*I think people need a lot more training on it [dementia], because it's something that is not to be frightened of.'* (Beth, daughter of couple who had accessed DA service) [(28), p. 389].

The second theme, incorporated findings concerning how the services promoted independence, through providing information directly and through signposting access to further support. As one care partner stated:

‘*It [the PSN] allows him to feel independent, and it allows me to be myself, or more myself*.' (Nancy, care partner from PSN site) [(28), p. 390].

The third theme illustrated how the PSN and DA service users considered that they had been enabled to establish a new, improved life with dementia. Self-esteem and self-worth were increased, and participants commented that they had been able to replace the social life and activities that they had lost due to dementia. One participant said:

‘*It's [the PSN] been the best thing that's happened to me for a few years now. I've been going to an art class for Alzheimer's and meeting people. It's fantastic because we can all talk to each other*.' (Lillian, person with dementia who had accessed PSN site) [(28), p. 391].

#### Visual Arts Enrichment Activities

The second study included in this review was conducted between 2013 and 2017 in the UK (29). This study aimed to evaluate the impact of visual arts enrichment activities (VAEA) on opportunities for the resilience of people with dementia. This study was part of a wider mixed methods study on dementia and imagination (30) that prioritized qualitative methodology. Papers reporting on the wider study were excluded from this review because they did not focus on resilience.

During the VAEA intervention, experienced participatory artists who had received training in dementia, used a person-centered approach to organize activities around the interests, abilities and energy of people with dementia (*n* = 48), aged 70–99 years, living in care homes (*n* = 4). The Clinical Dementia Rating scale (40) was used to rate the severity of participants' dementia. This found that the participants' dementia was borderline normal (*n* = 6), mild (*n* = 18), moderate (*n* = 8) and severe (*n* = 16).

The VAEA sessions lasted 2 h and were held weekly, for 3 months. The VAEA aimed to engage the senses of the participants in activities that could be, for example, individual collage painting or collective, film making, sculpture, or poetry. Participants also visited a contemporary arts center and a celebratory event was held that included their family and carers. Data was captured at 3 time points: baseline, when the activity sessions finished and 3 months after their cessation. Data was collected from people with dementia (*n* = 3) and family carers (*n* = 3) who were interviewed separately and the participatory artists (*n* = 2) who completed structured notes after each session. In addition, sessions were videoed, and recordings were observed to verify the study's findings. The data was analyzed in NVIVO, where multiple readings were used to identify emergent codes which were collated into themes.

This study found that the resilience of people with moderate and advanced dementia can be supported through VAEA. Newman et al. (29) found that VAEA provided a platform which facilitated creative expression; increased communication and self-esteem and that the intervention enhanced the relationships between participants with dementia, their carers and relatives. For example, collectively creating a poem relied upon participants expressing their emotional responses to their individual memories, of being at the sea. The first four lines of this poem were:

#### The Cruel Sea

The beautiful sea goddess

Godiva Pearl

Beautiful ruffles

*The ripples* [Poem Created by participants, (29), p. 8]

Creating the poem was facilitated by participants being of similar age and possessing compatible attitudes. Newman et al. (29) argue that in order to produce this adaptive response, participants drew on both personal and collective resources. These resources were cognitive, emotional, imaginative, and aspects of their social selves, including being able to perceive and interpret the thoughts and feelings of others in the group.

Participants were more resilient during the activity than they would have been without it. Newman et al. (29) describes how one person with dementia who was usually solitary and uncommunicative, was poised and passionate when painting. And, as a result of group singing, her interactions with others were observed to increase and be more socially engaging. Newman et al. (29) suggested that the VAEA increased her selfhood and therefore supported her resilience. A carer reported:

‘*It really did feel quite different to me all of the activities were bringing everybody together…. She was really connecting with other people as well in the group as well. Her whole body language seemed to be different.'* [Care home Director, (29), p. 11].

Self-esteem of participants was also increased, through participants' mastery of the activity and their success being praised by other people. However, self-esteem could also be undermined if a person was not able to accomplish the task or participate within the group and if the person's attention was drawn to their lack of ability and they became frustrated. Yet, when this occurred, participants demonstrated their ability to adapt because they still found the sessions enjoyable and wanted to participate in them. One man was able to participate, despite his communication difficulties, because he had developed a good relationship with the facilitators. It was argued that his resilience was supported through the social context of the VAEA.

Researchers also found that VAEA supported resilience through promoting personhood. VAEA enabled people to attain their potential without being inhibited by the assumptions other people made about their capabilities. In addition, VAEA increased the knowledge of carers and family members about the capabilities of people with dementia. One care home director said:

‘*I loved hearing people read and was surprised how confident the readers were. I suppose I'd underestimated how capable people with dementia are and had assumed they would find this difficult. You underestimate people don't you, you think ‘Oh they're not going to do that'*. [Care home director, (29), p. 13].

Improving the knowledge of significant others of the individuals' personhood meant VAEA had the potential to increase resilience in a sustained way in future interactions. This potential was also increased through VAEA giving residents, carers and relatives, an opportunity to celebrate and enjoy the activities together, in an atmosphere of positive equal relationships:

‘*It just felt like any social occasion/party-friends enjoying themselves, no distinction between those who were experiencing dementia and carers, family and friends.'* [Care home director, (29), p. 13].

### Memory Makers

The fourth intervention, “Memory Makers,” started in the USA in 2012 and was investigated in a study that aimed to explore its impact on resilience, using an observational descriptive study design (31, 32). This community-based intervention recruited people with dementia from memory clinics, medical practices and the Alzheimer's Association. To participate, people with dementia needed to be: in the early stage of their disease; aware of their diagnosis; able to discuss their feelings and experiences about dementia; have no behavioral psychiatric medical difficulties that would cause them to disrupt the group; have transport to the group and a care partner who was able to attend the majority of sessions. Participants included people with dementia (*n* = 35), aged 56–93 and family carers (*n* = 35).

“Memory Makers” provided structured education about dementia and psychosocial support in a group setting for people with dementia and their family carers. The groups were facilitated by master's educated social workers (two per group) who were trained with information from the Alzheimer's Association early stage group facilitators manual. Memory Maker sessions lasted 3 h and were conducted weekly for 8 weeks. During each session, people with dementia and carers (*n* = 12 dyads) were separated into two groups for 75 min, where they discussed different topics related to living with dementia. After this time, the groups joined. On the final session, the participants wrote a communal poem about their group bonding which aimed to capture the spirit of their resilience.

Data for the study was collected from consecutive groups (*n* = 4), at the end of each group of sessions, via an emailed online evaluation survey. This recorded perceived outcomes anecdotally. This study's findings, which will be described after the fifth intervention is introduced, were also derived from the facilitators' observations. Details as to how data analysis was conducted is not provided by the authors.

### Early-Stage and Beyond Community Activities

The fifth intervention was the Early-Stage and Beyond Community Activities (ESBCA) (31). This involved a range of activities (see Table 2) for people with dementia and family carers who were graduates from the Memory Maker program. ESBCA aimed to build resilience by developing community support. ESBCA was facilitated by trained social workers (31). Data was collected from family units (*n* = 1,799), that included people with dementia (*n* = 166), aged 49–93 years, and family carers (*n* = 178). The authors do not provide details as to how data was collected or analyzed.

The impact on resilience of the Memory Makers programme (32) and the ESBCA (31) will now be discussed together because the interventions involved similar participants and the findings of their investigations concur with one another. Matchar et al. (31) describes themes that were derived separately from people with dementia and their family carers. Here however, in keeping with the aims of this review only the themes identified for people with dementia will be reported. The eight themes identified were: acceptance; disclosure; significant others; sense of purpose; faith; routines, familiar environments, and memory aids; showing up/the value of a support group. The theme of acceptance relates to evidence in which participants described that they were resigned to having dementia, living with limitations and that they accepted this with a determination to make the best of life. One gentleman with dementia said:

‘*There's no changing it [having dementia]. I'm just rolling with it…. I want to find some strategy to best function…'* [Person with dementia, (31), p. 273].

Participants also identified that disclosure to others about their dementia was important to them as a source of support. This allowed them to continue with activities that they enjoyed. For example, one lady continued playing golf as her friends kept score for her. The second theme recognized that the support of significant others was crucial to people with dementia. Participants also highlighted the importance of having a sense of purpose and taking opportunities to stay engaged and socially active. Several participants adapted their activities to accommodate the dementia. Sometimes adaptation to continue activities occurred facilitated by friendships developed through Memory Makers. This happened when one person who could no longer drive was facilitated to continue with voluntary work, delivering donated food, because a Memory Maker friend, who also had dementia, drove them.

The theme, routines, familiar environments, and memory aids, revealed the ways in which participants benefitted from sharing strategies with one another. Doing so increased their knowledge and independence about managing daily life with dementia. Such strategies included keeping objects in the same place, keeping to the same routine including using the same shops or recreational facilities. The final theme illustrated clearly the beneficial impact of the Memory Makers group. Members valued attending the group. One person said it gave her “*renewed meaning*” in life [Person with dementia, Matchar et al. (31), p. 274]. Matchar et al. (31, 32) also reported that participants thoroughly enjoyed the “bubbly ambience” of Memory Maker, and ESBCA which were filled with fun, humor and laughter. One participant said:

‘*It's like a party…. Everyone's laughing, and everyone is happy'* [Participant with Dementia, (31), p. 274].

The atmosphere of the groups meant that participants could relax, be themselves, focus on their strengths rather than losses (32), in an environment which was free from stigma and one in which they felt safe to make mistakes (32). In the activities offered by both these interventions, participants were treated with “*acceptance, kindness, and respect*” and the study authors argue that this helped participants to build and maintain their resilience (31). One participant illustrated these findings saying:

‘*Everyone in the group ‘got it' and that was a very liberating experience……I felt less like complaining and more inclined towards positive planning and living one day at a time'* [Person with dementia, (32), p. 174].

Being a member of the group provided participants with a sense of belonging. One participant said they had gained a new family, and this empowered them as individuals. The power of the group and the bonds created within them was captured in a poem that participants created:

‘*You are not alone*.

I felt the group was a life saver

*It brought a life, empowering us all'* [(32), p. 173].

Key features of the studies and interventions are summarized in Table 2.

Having provided an overview description of the studies, this paper now focuses on how the concept of resilience was defined and operationalized during the investigations.

## Definition and Operationalization of Resilience

To address the aims of this review to fully examine and integrate the findings of the studies it is important to establish how resilience was defined and operationalized. This is because historically resilience has been defined in different ways (8). Newman et al. (29) and Matchar et al. (31, 32) state that they defined resilience as a dynamic process that encompasses positive adaptation in the presence of adversity. Although a definition of resilience is not expressly stated by Clarke et al. (28), the study's design supports the supposition that resilience was defined as a process. This is because resilience is reported to be an outcome of the DA and PSN interventions, and yet quantitative outcome measures of well-being and quality of life are used in the study, rather than scales that aim to measure the psychological capacity for resilience.

The studies identified in this review were informed by different research paradigms and theoretical backgrounds including, social constructivism and social disability (28); the ecological theory of human development and the ecological framework of resilience (29); and the social context perspective (31, 32). Newman et al. (29) explicitly stated that the ecological view of resilience underpins their investigation, but all the studies included in this review appear to endorse the view that the resilience of people with dementia is impacted by resources that are accessed at individual, social and community level (8). This supposition is supported because the interventions target both people with dementia and their family carers and the wider community.

Because resilience has been operationalized in this way it is appropriate to apply the resilience framework (9) to the studies' findings to facilitate a more in depth examination as to how the interventions impacted the resilience of people with dementia. This is relevant because resilience can potentially be supported through: reducing the adversity and via improving the provision or access to resources. In applying the framework in this review, community level resources that support resilience are defined as being people in direct contact with people with dementia (significant others) and societal level resources are defined as referring to people outside immediate contact with individual service users.

The results of applying the framework to the included studies are discussed below and summarized in Table 3.

**Table 3:** The impact of interventions applied to the Resilience Process (8) and Framework (9).

	**Adversity**	**Resources**	**Outcomes for individuals diagnosed**
PSN and DA Services Clarke et al. (27, 28)	Identified and address a wide range of needs.	**Individual** - Provided resources relevant to strengths and desires of individuals. e.g., people wanted to keep well, wide range of services, and purposeful activities - Supported access to resources, through empowering participation, choice, independence, control. **Community** - Social contact with peers - Supported significant others. **Societal** Education for others in society and advocated on behalf of people with dementia.	- Increase QoL, independence. - Achieved a “new normal” living with dementia. - Recommenced social life and purposeful activities. - Improved self-esteem, self-worth, improved self- identity, confidence to disclose dementia to others.
Memory Maker and ESBCA Matcher et al. (31, 32)	Identified Stigma Reduced Social Isolation	**Individual** - Access to information about successfully living with dementia **Community** - Opportunities to support and receive support from others - Social contact with peers. - Opportunities for social life and environment without stigma.	- Improved independence, positivity,communication. - Adapted purposeful activities. - Empowered to seek further help through disclosure. - Reframed dementia normalizing existence.
VAEA Newman et al. (29)	Variable cognitive and communication difficulties, Stigma Excessive Disability	Access to group and individual creative activities Access to context which supports personhood. Access to and use of interplay of individual and social resources.	Did better than expected. Increased communication, self- esteem Improved relationships with others.

## The Impact of the Five Interventions on the Resilience of People With Dementia

### DA and PSN Services (27, 28)

The DA and PSN services supported resilience by helping to identify the adversity and needs of people with dementia. This included identifying participants' needs and desires to have a wide range of activities to help them stay well. The adversity experienced by individuals through the stigma of dementia was also combated through DA facilitators providing education to groups of people (other than the participants) about dementia and the needs of people with dementia.

The interventions enabled access to resources that occurred at individual, community and societal levels. Individual resources included the activities that were applicable to people's individual strengths, needs and desires. Indeed, access to these was supported through the participants' increased independence and sense of control. One participant referred to how the services empowered her make choices with her partner:

‘*It gave us the confidence to move in the directions we wanted to move in'* (Jilly, care partner who had accessed DA service) [(27), p. 392].

At community level, the interventions impacted the resilience of the people with dementia through providing support to their carer and through providing participants with access to social peer support. Lillian, a participant with dementia said of the PNS:

‘*It's fantastic because we can all talk to each other'* [(27), p. 391].

Having access to resources appeared to impact the outcomes of resilience. The theme, “getting a life back” speaks to participants having achieved a “new normal” and improved quality of life. One of the participants said:

‘*We've sort of got back some normality now. He's got quite a week of things happening most days*.' (Carer) [(27), p. 391].

### Memory Maker and ESBCA (31, 32)

The Memory Maker and ESBCA, did not use a specific tool to identify the needs of the people with dementia as part of the investigation. Never-the-less, it can be extrapolated from the study's data, that participants were experiencing adversity particularly regarding social isolation as a result of the dementia and stigma.

The interventions provided participants with time with others who shared their experiences as people with dementia and family carers. Having time to bond as a group was a resource for individuals and the community through which resilience could be supported and sustained (31).

The outcomes of these interventions for resilience, were improved communication between people with dementia and care partner dyads (32), increased capacity for empowerment, independence, and positivity going forward into the future. The data also suggested a more global outcome, that group membership helped move individuals toward a more normal life with dementia, which included being themselves and having a social life with friendships that reduced social isolation. In this regard, their lives with dementia were normalized and the dementia was reframed as being part of their lives. The findings further revealed that participants had more confidence to disclose their dementia to other people (31). This suggests that not only do these outcomes have the potential to be sustained within this community of participants, but outcomes could potentially develop as a result of individuals seeking and benefiting from the support of others outside this immediate peer group community. However, evidence that this occurred is not provided by Matchar et al. (31, 32).

### VAEA (29)

In terms of reducing adversities, the VAEA intervention highlighted that participants had cognitive and communication difficulties, that were more severe than those experienced by participants in the other studies. The severity of difficulties was variable both in and between individuals (29). Newman et al. (29) also identified that the beliefs and actions of carers and relatives, regarding the person with dementia's capabilities, impacted how adversity was experienced by people with dementia. Newman et al. (29) found that seeing people with dementia involved in VAEA increased their awareness. This could potentially change the behavior of carers and relatives resulting in them acting in way that supported resilience and did not cause excessive disability. However, no evidence of this change was reported by the study.

In contrast to the other studies, Newman et al. (29) argue that participating in the VAEA increased access to resources but these resources could not be separated into distinct individual and community categories. Instead, resources were used in a complex interplay which was enabled by VAEA. VAEA had no visible impact on resilience through wider societal issues but the impact was through individual, and community issues as described above.

VAEA resulted in people doing better than would otherwise be expected and this can be regarded as an outcome of resilience, during the intervention. In addition, their communication and interaction with others increased in quality and their self-esteem improved.

The findings of this review are now discussed in relation to the wider literature and then recommendations for future research are proposed.

## Discussion

The studies reported the perceptions and experiences of people with dementia and the findings reveal that the interventions were well-received by participants who engaged with them voluntarily. Many people with dementia reported the interventions to be beneficial and their views concurred with the observations and opinions of significant others (27, 28, 31, 32). Newman et al. (29) reported the experiences of people with dementia using the intervention as being beneficial to their resilience but did so using the observations and verbal reports of significant others, rather than directly from people with dementia. This raises questions about the challenges involved in assessing the resilience of people with moderate and advanced dementia. As dementia progresses it is important to find ways to accurately capture the perspectives of people with dementia about their resilience. Not to do so is potentially problematic because the perspectives of people with dementia and carers can differ regarding perceptions of quality of life (41) and what makes activities meaningful (42).

One of the benefits of the interventions, was that they empowered people with dementia to disclose their diagnosis to other people (27, 28, 32). Disclosure of dementia diagnosis to friends and family is beneficial (43) and it is logical that informing significant others may be a gateway to the person gaining support from significant others. This finding was less pertinent in the study population living in residential care, but it is notable that involvement with VAEA also improved communication with other people (29). The latter could improve the possibility of compensatory support which may increase the resilience of people with dementia (4).

It is noticeable that only the study reporting VAEA described any weakness or disadvantages to the interventions. VAEA was reported as enjoyable despite some people with dementia experiencing frustration, if they were unable to master certain activities.

The results of this review reveal that most studies to date have focused on people with dementia who are “doing okay” (44). Participants with dementia who were recruited for Memory Makers and ESBCA were relatively well-supported, and those accessing PSN and DA services had the capacity to reach out to the services and engage with them. Although participants involved with VAEA all had significant vulnerabilities, only people without severe communication difficulties were involved in the study. Clarke et al. (28) acknowledges that not accessing people with dementia who did not use the service, was a limitation of their investigation. In addition, except for some participants, involved with VAEA, most participants had early stage dementia. Therefore, the findings of the community-based studies reflect the impact of the interventions on the resilience of people with dementia who have a relatively high ability to access and use resources to support their resilience. This is a situation common to other studies conducted regarding resilience in people with dementia where participants were deemed to be “doing okay” (15, 44), living with people who were supportive and willing to participate in research (22, 45, 46), had contact with support groups (16, 47, 48), and were in receipt of support services (19). However, Harris (44) investigated the resilience of people with dementia including some who were not “doing okay” (*n* = 5). Therefore, it is possible to examine the resilience with people with dementia who are adapting less well to the challenges of living with dementia. Accessing and recruiting participants who are in the most need can be challenging (49). It may take more time to convince gatekeepers that such individuals would be able to participate and to gain participants' consent (50). It may also be challenging to convince funders that recruitment time and study duration in the context of dementia research may need to be extended to facilitate the inclusion of individuals who are in most need.

This review identified that a small number of studies have examined interventions that aimed to support the resilience of people with dementia, who live in both community and residential care settings. The studies were undertaken within the last decade and three of the papers reporting their evaluations were published in 2018. This suggests that the investigation of interventions to support resilience in dementia is a relatively recent and developing field of research and practice. This novelty is reflected in the research designs used to evaluate the interventions. The assessment of the studies methodological strengths and weaknesses during this review found that all the studies produced valuable results in terms of their contribution to knowledge and regarding the aims of this review. However, only Clarke et al. (27, 28) and Newman et al. (29, 30) rated highly in terms of methodological quality. This result was obtained by the two reviewers whose independent assessments, which initially revealed a high degree of consensus, achieved full consensus following discussion (Appendix ii in Supplementary Material).

The research designs of all the studies do not seek to measure change in well-being but instead seek to describe how the services were used and experienced by people with dementia and to identify what stakeholders perceived their impact to be. Matchar et al. (31, 32) and Newman et al. (29) focused on describing details of the perceived process and outcomes of the interventions. Their investigations infer that outcomes are as a result of the interventions, and there was no attempt to isolate variables and measure change. Newman et al. (29) did obtain data at multiple time points in relation to participation in VAEA, including data obtained 3 months after the intervention, but their findings concerning potential changes in well-being beyond the VAEA sessions were not reported. Without alternative study designs providing control group comparisons, it is impossible to ascertain effectiveness and whether participation was beneficial due to the components of each intervention per say, or due to them being offered in the absence of another viable activity. Therefore, it cannot be determined to what degree the social component of the group interventions were important. Never-the-less, it should be noted that the varied interventions examined here all supported resilience through socially related characteristics namely, their positive impact on stigma, social contact, and social support.

The stigma associated with dementia was highlighted as an adversity in that it contributed to excessive disability (29) and social isolation (28, 31, 32). This concurs with findings elsewhere, that the actions of other people in applying negative stereotypes increase the difficulties of living with dementia (43). It is therefore significant that the interventions reduced these adversities through providing stigma free, psychosocially safe platforms (29) in which people were free from the fear of potential embarrassment (43, 51).

Social contact and support from other people are also important for resilience (5, 15, 22), as is the quality of the relationships that people with dementia have with other people (52). It appears that the quality of relationships within all the group interventions were improved because they involved people who shared the experience of living with dementia. The interventions provided the opportunity for participants to interact and belong to a peer group and this was hugely valued by participants (28, 29, 31, 32). Belonging to a peer group is known to positively impact resilience in dementia (16, 19, 53) by empowering people, providing opportunities to share practical information strategies to increase their repertoire of adaptive coping strategies (54) and enhancing positivity, which is important for resilience (15, 55).

The quality of relationships between people with dementia and the interventions' facilitators were also important to the success of the interventions. It is notable that all the interventions involved facilitators who played key roles conducting and creating both the content of the interventions and their processes. The artists created the VAEA intervention, enabled participation and ameliorated the impact on self-esteem for individuals who were not able to master the activity (29). The lay health advisors of the DA intervention had ongoing in-depth interactions with service users throughout their journeys with dementia and they shaped the service in response to needs (27, 28). In order to be effective, these facilitators had direct personal knowledge of dementia and intimate knowledge of communities (27) and had training in dementia care as social workers (31) or as researchers (29). This reveals that successful resilience building interventions requires skilled facilitation. In addition, as dementia progresses the way facilitators facilitate interventions is likely to differ and require additional skills.

The interventions also impact resilience through being supportive of the personhood (56) of individuals with dementia, by providing them with meaningful activities (29, 31). Meaningful activities are likely to contribute to the increased self-worth and self-esteem that resulted from the interventions (27–29). Indeed, the activities may support resilience through providing a sense of continuity in identity (55, 57) which can be balanced against the changing perceptions of identity that occur due to the dementia. Successfully managing this balance is important for resilience in dementia (53, 58). The proposition that the interventions may support this important “task” of resilience in dementia is supported by participants in the studies who said the interventions helped them adjust to dementia, to reframe and normalize living with the condition (27, 31, 32).

Another characteristic of the interventions is that they were strength-based in that they built upon the assets and resources that people with dementia already possessed. The interventions required participants to have and use personal and social skills in order to participate. For example, the group interventions required communication and cooperation skills and when participating in the VAEA interventions, participants used aspects of their personal and shared cultural, previous and present identities (29). This implies that people with dementia did not just receive the interventions, but they contributed of themselves, to the intervention and to other group members. Indeed, because their participation involved reciprocity, questions can be raised as to how the positive impact on the resilience of individuals might be increased through the interventions providing opportunities to contribute to communal resilience. People with dementia want to give support to others and contribute (16, 59, 60) and doing so provides them with opportunities for increased self-worth through reinforcing positive self-identity (43). It appears that when the interventions impacted as resources to support resilience, the lines between individual and community resources are blurred regarding reciprocity. Lines between these resource categories were also blurred because two out of three interventions aimed to support the resilience of both people with dementia and family carers. Indeed, carers can be regarded as a community support for people with dementia and family carers with greater well-being may have greater capacity to support the resilience of the people with dementia. It is also noteworthy that Newman et al. (29) identified there was interplay between individual and community resources during VAEA.

The findings of the review revealed that resilience can be fostered “in the moment” during an intervention and/or sustained after the intervention has finished. “In the moment” increased adaptation was highlighted during VAEA, whereas the community-based interventions placed greater emphasis on supporting resilience with the goal of attaining sustainable outcomes (31), through increased independence and on-going well-being (27, 28). However, fostering of resilience “in the moment” was implied during all the interventions through the reports of humor, joy, and release reported by participants. These positive emotions equate to what have been described as “good moments” (61) of happiness. It appears logical that interventions that create opportunities for small moments of happiness are likely to increase positivity and hope which are very important for resilience in people with dementia (16, 19, 21).

The studies in this review provide some evidence that the effects of interventions on resilience can be sustained. Some people with dementia reported and recalled the effects of the interventions when data was obtained (27, 28) and after they had occurred (32) and effects of ESBCA were on-going (31). On-going sustained effect was not highlighted in the findings of VAEA, but the potential for this exists if carers were to act differently due to improvements in relationships and increased awareness concerning the capabilities of people with dementia. Potential for sustained effect on resilience also exists regarding the other interventions due to their empowerment effects and the potential for increased support from other people due to disclosure.

## Implications for Future Research

Applying the resilience process (8) and framework (9) to the interventions discussed in this review, facilitated in-depth understanding as to how these interventions impacted resilience of people with dementia. Therefore, it would be useful for future research to include a resilience perspective using the framework. Doing so would be particularly beneficial to investigations concerning interventions that aim to support people with dementia in any purposeful activity. This is because purposeful activity, chosen by a person (57, 62) and compatible with their tacit norms (63), is important to the resilience of people with dementia (48, 55, 57, 63, 64). For example, spirituality can be an important resource for the resilience of many people with dementia (5, 19, 48, 51, 59, 65–67). But to date interventions targeting spirituality have not, to our knowledge, focused on resilience even through spirituality based interventions have been found to impact well-being (68–70). Applying the resilience process when examining such interventions may increase understanding of how they impact well-being.

This review also found that although supporting resilience was beneficial to people with dementia, only a limited number of interventions have been developed and these have been assessed predominantly with people in early dementia in community settings who are currently “doing okay.” Future research should focus on interventions that have the potential to support resilience through facilitating communication opportunities for people with more advanced dementia to interact with other people (58, 71, 72). Indeed, touchscreen technology has been found to enhance personhood of people with dementia (73) and robotic technologies can positively impact quality of life (74, 75) and improve mood (76). The potential of these interventions to support resilience could be investigated. Indeed, focusing on people with moderate dementia and those not “doing okay” might reveal the need to target different areas and develop different strategies to support their resilience.

This review highlights gaps in current knowledge concerning how interventions support the resilience of people with dementia and their carers differently, similarly, and jointly. This warrants further investigation, as joint interventions may not suit all dyads and could even harm the resilience of either party. Furthermore, if it is found that the resilience of people with dementia can be enhanced by interventions that also support family carers, then the potential for interventions that jointly target the resilience of people with dementia and professional carers should be investigated in residential care settings.

The findings of this review suggest that resilience can be supported “in the moment” and/or sustained after interventions. This suggests that resilience in relation to time needs further examination. Further investigation is also warranted concerning the interplay between individual and community resources (29), particularly regarding reciprocity and how reciprocity can be used in interventions to support resilience in people with dementia.

It is difficult assess the impact of interventions that occur in clinical environments (77), particularly when investigating them in the context of dementia, which is a progressive disease and where the symptoms of the disease and the adversity caused vary within and between individuals (29). Therefore, in order to potentially influence policy and practice, future research ideally needs to use methodologies that elucidate changes that occur both during and as a result of interventions. Furthermore, tools such as Dementia Care Mapping (78) and the Observational measurement of Engagement (79), may be needed to accurately capture behavioral responses to stimuli and measure changes in resilience. In addition, because the resilience of individuals is impacted by significant others, it is important that future investigations examine the social context into which interventions are introduced and their impact on resilience in the light of this.

## Limitations

This review has limitations and its findings should be considered in the light of these. The search was limited to items published in English and in order to focus on resilience, it excluded proxy terms for resilience. Therefore, intervention investigations reported in different languages and those that explored alternative well-being outcomes, which may have impacted resilience, were omitted. In addition, the search process and data extraction were conducted by one reviewer therefore some relevant articles might have been erroneously excluded. However, the review utilized a theoretically informed systematic approach and the included studies were subjected to in-depth analysis applying resilience theoretical constructs.

## Summary

This review used a systematic approach to identify and examine research that investigated psychosocial interventions that aimed to support the resilience of people with dementia. The findings revealed a variety of interventions conducted in both residential care and community living settings. The interventions were found to impact all the components of the resilience process (8) and sometimes there was interplay between the individual and social resource components of resilience. The findings reveal that interventions can support resilience, both during and after the intervention sessions, although evidence of their effectiveness is limited because studies are descriptive and do not measure change. This review found there is a need for further research in this developing field. However, interventions that successfully build resilience in people with dementia need skilled facilitators to ensure that they are supportive of personhood and that they enable reciprocal social interactions to occur. It is also important that interventions are provided within a stigma-free context.

## Author Contributions

SW conceived, planned, and conducted all aspects of the review including writing the manuscript. ÁT conducted critical appraisal of the included papers, reviewed the drafts, and the final paper. DC provided guidance, critically reviewed drafts, and the final paper.

### Conflict of Interest

The authors declare that the research was conducted in the absence of any commercial or financial relationships that could be construed as a potential conflict of interest.
