# Supplementary figures and images for: Fostering the Resilience of People With Dementia: A Narrative Literature Review
Source: Front Med (Lausanne). 2020 Feb 25;7:45. doi: 10.3389/fmed.2020.00045 (PMC7051935; doi:10.3389/fmed.2020.00045)

# Appendix i: Example of search Strategy from Cinahl Database, EBSCOhost


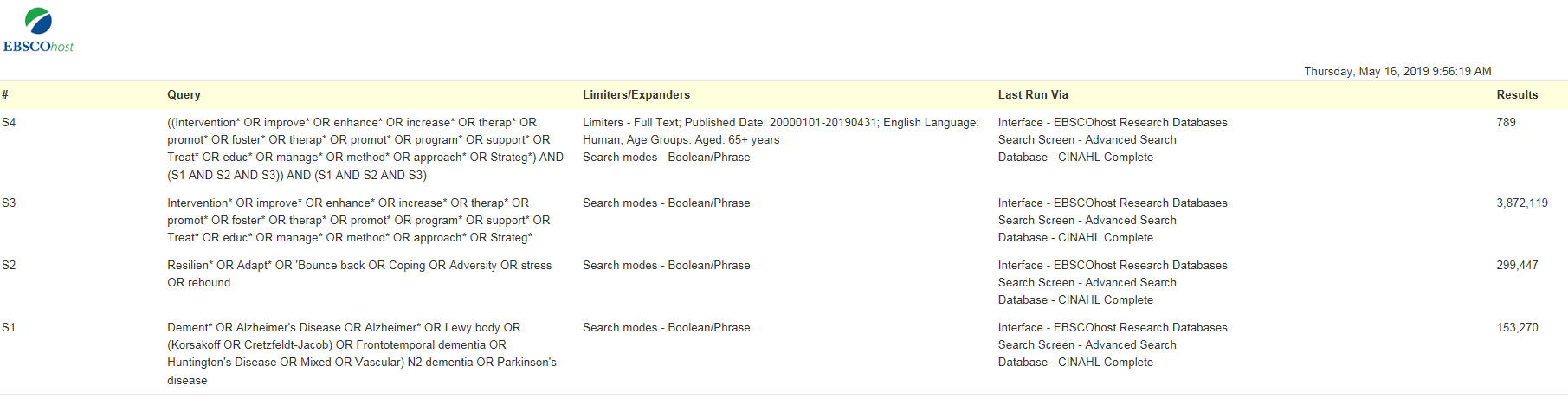

Supplement: Supplementary file 1 [file Table_1.DOCX]
