# Supplementary material for: Fostering the Resilience of People With Dementia: A Narrative Literature Review
Source: Front Med (Lausanne). 2020 Feb 25;7:45. doi: 10.3389/fmed.2020.00045 (PMC7051935; doi:10.3389/fmed.2020.00045)
Supplement: Supplementary file 2 [file Table_2.DOCX]

# Appendix II: Results of Quality Appraisal
